# Evaluation of urine glutathione peroxidase 4 in cats with chronic kidney disease

**DOI:** 10.3389/fvets.2025.1756038

**Published:** 2026-01-14

**Authors:** Wei-Li Hsu, Sheng-Hui Huang, Zeng-Yei Hseu, Vin-Cent Wu, Ying-Hao Chen, Ya-Jane Lee

**Affiliations:** 1 Graduate Institute of Microbiology and Public Health, College of Veterinary Medicine, National Chung-Hsing University, Taichung, Taiwan; 2 Institute of Veterinary Clinical Science, School of Veterinary Medicine, College of Bio-Resources and Agriculture, National Taiwan University, Taipei, Taiwan; 3 Department of Agricultural Chemistry, National Taiwan University, Taipei, Taiwan; 4 Department of Internal Medicine, National Taiwan University Hospital, Taipei, Taiwan; 5 Department of Veterinary Medicine, School of Veterinary Medicine, National Taiwan University, Taipei, Taiwan; 6 National Taiwan University Veterinary Hospital, College of Bio-Resources and Agriculture, National Taiwan University, Taipei, Taiwan

**Keywords:** feline, ferroptosis, progression, renal disease, urine biomarkers

## Abstract

**Introduction:**

Ferroptosis is a distinct form of regulated cell death characterized by iron-dependent lipid peroxidation that damages cellular membranes and leads to the end of a cell’s life. Glutathione peroxidase 4 (GPX4), the only enzyme capable of the reduction of lipid peroxidation products within cells, is a key regulator of this process.

**Aims:**

The role of GPX4 in feline chronic kidney disease (CKD) has not been previously investigated. This study aims to determine whether urine GPX4 levels are associated with CKD severity in cats and to assess their potential as a progression biomarker.

**Methods:**

Urine GPX4 levels were measured using a commercial feline ELISA kit. The urine-GPX4-to-creatinine ratio (UGCR) was calculated. Fifteen healthy cats, 61 cats with CKD, and six cats with acute-on-chronic kidney disease (ACKD) were included in the study.

**Results:**

Compared with the control group (urine GPX4, median [IQR]: 25.21 [18.99–26.91]; UGCR: 0.072 [0.057–0.101] × 10^−4^) and the early-stage CKD group (urine GPX4: 24.31 [22.00–24.07]; UGCR: 0.134 [0.070–0.260] × 10^−4^), cats with late-stage CKD showed significantly higher levels of urine GPX4 (26.89 [25.11–31.66]; *p* = 0.011) and UGCR values (0.271 [0.197–0.457] × 10^−4^; *p* < 0.001). Within the CKD subgroups, UGCR was significantly higher in cats with proteinuria, hypertension, anemia, and those receiving iron supplementation (all *p* < 0.003). Serum creatinine levels and WBC counts were identified as independent variables that were correlated with UGCR. Cats in the CKD progression group had higher UGCR than non-progressors, and an elevated UGCR was associated with an increased risk of CKD progression (hazard ratio [HR], 1.75; 95% CI, 1.20–2.54; *p* = 0.003).

**Conclusion and clinical importance:**

UGCR increased with the severity of CKD and was significantly associated with serum creatinine concentration and disease progression. Urine GPX4 may thus serve as a novel biomarker for monitoring renal deterioration and progression in cats with CKD.

## 1 Introduction

Several cell death pathways contribute to the development and progression of both acute kidney injury and chronic kidney disease (1). Among these pathways, ferroptosis can be distinguished by its dependence on iron-driven lipid peroxidation that ultimately leads to oxidative damage to cell membranes, which then drives the pathogenesis of multiple disease states (2). Ferroptosis is a distinct form of regulated cell death and is different from apoptosis, autophagy, and necrosis. Morphologically, it is characterized by shrunken mitochondria, increased membrane density, and a reduced or absent of mitochondrial cristae; this is without any of the nuclear changes that are typically associated with apoptosis (3, 4). Accumulating evidence indicates that ferroptosis is implicated in various forms of pathological cell death, including ischemia–reperfusion injury (5, 6), neurodegenerative disease (7, 8) and cancer cell death (9, 10).

Ferroptosis is primarily linked to an overloading of intracellular iron that facilitates lipid peroxidation (1). In addition to abnormalities in iron metabolism, the inhibition of intracellular antioxidant defenses can also trigger ferroptosis. Under physiological conditions, a transmembrane protein complex known as system xc^−^, mediates the exchange of extracellular cystine and intracellular glutamate across the plasma membrane, thereby supporting the biosynthesis of glutathione peroxidase 4 (GPX4) (3). This process is critical to glutathione production and oxidative protection within cells, as lipid peroxides are able to be metabolized by GPX4-catalyzed reduction reaction (4, 11–13). Consequently, GPX4 serves as a central regulator of ferroptosis.

Proper kidney integrity depends on the presence of functional GPX4 protein (12). In an inducible GPX4-depleted mouse model, massive renal tubule cell death and acute kidney injury were observed within 2 weeks (12). In addition, kidney tubular cells are perhaps the most ferroptosis-sensitive cells in mammals (12). Ferroptosis has been shown to be a key driver of both cisplatin-induced acute kidney injury (AKI) (14), and ischemia–reperfusion–associated renal necrosis (5). Emerging evidence suggests that this pathway is involved in the progression of chronic kidney disease (CKD) in humans (15). A decreased abundance of GPX4 has been observed in renal tubular cells of CKD patients (16), as well as in murine models of renal fibrosis that were induced by unilateral ureteral obstruction (UUO) (16, 17) and ischemia–reperfusion injury (18, 19). Inhibition of ferroptosis has been shown to attenuate kidney injury and fibrosis in various CKD animal models (16–18, 20). Furthermore, GPX4 expression has been proposed as a potential predictor of disease progression in diabetic kidney disease (21).

Despite these advances involving human and experimental models, the investigation of the role of ferroptosis in veterinary CKD is still largely unexplored. Accordingly, the present study evaluated urine GPX4 levels in cats across different stages of CKD and acute-on-chronic kidney disease (ACKD) to determine whether there is any association with disease severity and to explore the potential involvement of ferroptosis in renal deterioration.

## 2 Materials and methods

### 2.1 Animals

Retrospectively, we enrolled feline patients presenting with an elevated renal index or that were undergoing routine health examinations at the National Taiwan University Veterinary Hospital between September 2019 and March 2022. The patients were classified into the control group, the CKD group, and the ACKD group, based on clinical assessment.

The control group contains cats without any clinical signs or abnormal physical examination results. These cats also had no medical history of renal-related, neuro-related, cardio-related, oncology-related, or endocrine-related symptoms. No medication was administered to the animals at the sample collection time point, except for the monthly heartworm and ectoparasite prevention. In addition to normal blood urea nitrogen (BUN, <30 mg/dL) and creatinine concentration (<1.6 mg/dL) being measured as part of their routine blood examination, other parameters were assessed, namely SDMA, hematocrit, red blood cell counts (RBC), white blood cells counts (WBC), plasma alanine aminotransferase level (ALT), plasma aspartate transaminase level (AST), plasma alkaline phosphatase level (ALKP), plasma albumin level (Alb), total protein (TP), plasma glucose level, plasma sodium level, plasma potassium level, and plasma chloride level, and these needed to be within the appropriate reference ranges for a cat to be included in the control group. Additionally, the results of an abdominal X-ray, urinalysis, and urinary tract ultrasound revealed no significant findings for the control group cats.

Cats classified into the chronic kidney disease (CKD) group met at least one of the following criteria. Firstly, persistent azotemia, defined as a plasma creatinine concentration ≥1.6 mg/dL for a duration of at least 3 months. Secondly, persistent proteinuria, defined as a urine protein-to-creatinine ratio (UPC) > 0.4 on at least three occasions, each separated by more than 2 weeks. Finally, radiological abnormalities consistent with CKD, such as small, irregular kidneys, reduced corticomedullary distinction, and/or polycystic kidney disease.

**Figure 1:**
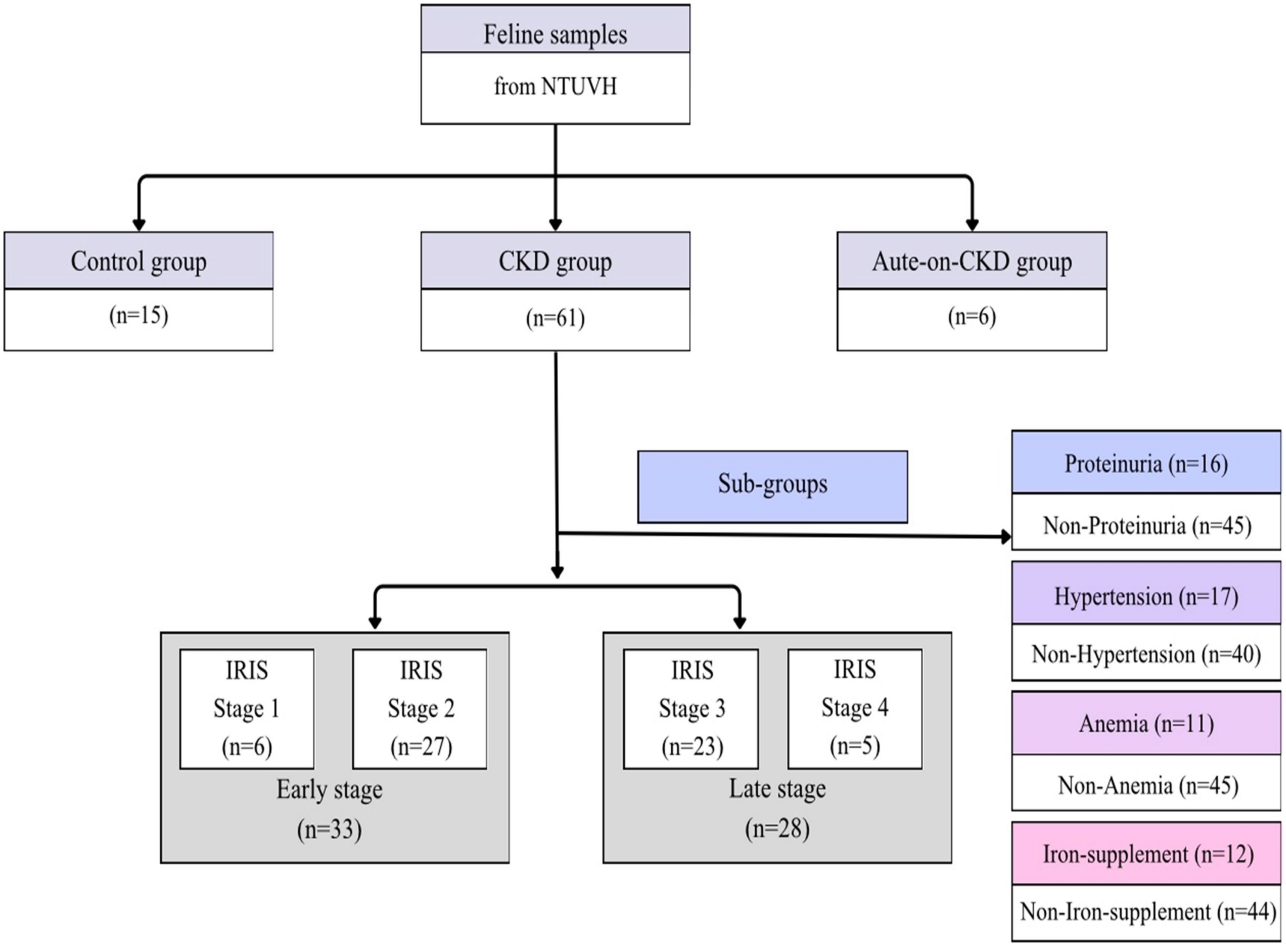
Grouping algorithm and case numbers for each study group.

All CKD cases were staged according to the International Renal Interest Society (IRIS) staging system (Figure 1) into stages 1–4. For each case, initial symptoms, history, physical examination findings, diagnostic imaging results, and clinicopathological data were recorded. Cats diagnosed with neoplasia, endocrine disorders, or systemic infectious diseases, such as, blood parasites or viral infections, including feline leukemia virus (FeLV), Feline Immunodeficiency Virus (FIV) or feline infectious peritonitis (FIP), urinary tract infection (UTI), or post-renal azotemia (e.g., ureteral or urethral obstruction), when confirmed by ultrasonographic renal pelvic dilatation (>0.3 cm), were excluded.

Cats were categorized into subgroups based on the presence of CKD-related complications, including proteinuria, hypertension, anemia, and iron supplementation. Proteinuria was defined as a urine protein-to-creatinine ratio (UPC) > 0.4 on three separate occasions at least 2 weeks apart. Hypertension was defined as systolic blood pressure > 180 mmHg on two separate measurements or by the documented administration of amlodipine. Anemia was diagnosed when at least two of the following three hematologic parameters were below the reference range: hematocrit, hemoglobin, or red blood cell count. Cats that had received oral or intramuscular iron supplementation for a minimum of 1 month were classified into the iron supplementation group.

To assess factors associated with CKD progression, cats were further divided into two groups: progression and non-progression. Progression was defined as a 15% or greater increase in plasma creatinine concentration from baseline (22) over a 180-day follow-up period.

### 2.2 Sample collection and storage

Blood was collected through venipuncture of the jugular or cephalic vein. Serum samples were obtained from tubes containing a clot activator. The samples were next centrifuged at 1,500×*g* for 3 min, and then the supernatants were stored at −80 °C until further analysis. Hematological values were analyzed using the Exigo hematology analyzer and the IDEXX Procyte Dx* hematology analyzer. Plasma chemistry profiles were measured using the Ortho Clinical Diagnostics™ VITROS™ 350 system. Plasma electrolytes were determined using the Roche Cobas b 121 system. Urinalysis was performed using the Roche Cobas b 411 system.

### 2.3 Measurement of urine GPX4 by feline commercial GPX4 ELISA kit

Urine GPX4 levels in the cats were measured using a species-specific commercial ELISA kit (MBS093863, MyBioSource, USA) by following the manufacturer’s instructions. Standards and urine samples were tested in duplicate. After adding the HRP-conjugate reagent, plates were incubated at 37 °C for 1 h. The wells were then washed four times with 1× wash buffer. Finally chromogenic substrates (50 μL each of Substrate/Chroomogen A and B) were added sequentially, followed by incubation at 37 °C for 15–20 min while protected from light. The reactions were terminated with 50 μL of Stop Solution, and absorbance was read at 450 nm within 15 min using a microplate reader. GPX4 concentrations were derived from the relevant standard curves. According to the manufacturer’s datasheets, the feline GPX4 ELISA kit has a sensitivity of 1.0 ng/mL and a detection range of 3–100 ng/mL or 12–100 ng/mL, with intra-assay or inter-assay coefficients of variation (CVs) of less than 15% (Supplementary Table S1). The urine-GPX4-to-creatinine ratio (UGCR) was also measured.

### 2.4 Statistical analysis

We used R (version 4.1.2, R Foundation for Statistical Computing, Vienna, Austria) for data analysis and figure plotting. Numeric continuous variables were evaluated for normality through visual inspection of histograms and by the Shapiro–Wilk test. Most data were not normally distributed; therefore, for consistency, all numerical data are presented as medians with interquartile ranges (25th–75th percentiles). The Wilcoxon rank-sum test was used to compare parameters between two groups. Kruskal-Wallis tests, followed by Dunn’s multiple comparison tests, were employed for comparisons among three or more groups. The Chi-square test or Fisher’s exact test was performed to assess associations between categorical variables. Simple linear regression and multivariate linear regression analyses were conducted to evaluate the associations between UGCR and clinical parameters. Multivariate linear regression analyses were performed using a backward stepwise approach that including variables with a *p*-value less than 0.05. Strength of association was interpreted using *R*^2^ values with 0.1 approximately, indicating a weak association, 0.2–0.3, indicating a moderate association, and ≥0.4, indicating a strong association.

To identify predictors of CKD progression, Cox proportional hazards regression analysis was conducted to estimate the hazard ratios of clinical parameters linked to disease progression. Receiver operating characteristic (ROC) analysis was conducted to assess the ability of the urine GPX4-to-creatinine ratio (UGCR) to discriminate cats that experienced CKD progression within a 180-day follow-up period, and area under the ROC curve (AUROC) was compared with the null hypothesis of an AUROC of 0.5. Additionally, the optimal cut-off value was determined using maximally selected rank statistics (surv_cutpoint function, R package *survminer*), which identifies the threshold that maximizes the standardized log-rank statistic while accounting for multiple testing. Based on this cut-off value, CKD cats were stratified into groups and compared using Kaplan–Meier survival analysis with the log-rank test. A *p*-value < 0.05 was considered statistically significant.

## 3 Results

### 3.1 The performance characteristics of the ELISA kits

Standard calibration curves were successfully established. The calibration curves were generated using five concentrations (3.12, 6.25, 12.5, 50, and 100 ng/mL), and demonstrated excellent linearity within the manufacturer’s specified detection range (*R*^2^ = 0.9998). A representative standard curve is shown in Supplementary Figure S1. All duplicate measurements showed intra-assay coefficients of variation below 15% (12.7%), confirming acceptable repeatability.

### 3.2 Study population

A total of 82 cats were enrolled in this study, including 15 healthy cats, 61 CKD cats and 6 ACKD cats. Among CKD subgroups, there were 6 cats in the IRIS stage 1 subgroup, 27 cats in the IRIS stage 2 subgroup, 23 cats in the IRIS stage 3 subgroup, 5 cats in the IRIS stage 4 subgroup, and 6 cats in the ACKD subgroup. Because of the small size of IRIS stage groupings, the cats were also separated into an early-stage group (IRIS stages 1 and 2) and a late stage group (IRIS stages 3 and 4), which contained 33 and 28 cats, respectively (Figure 1).

In the control group, the most common breed was domestic shorthair (*n* = 12), followed by American shorthair (*n* = 1), British shorthair (*n* = 1), and Ragdoll (*n* = 1). In the CKD group, domestic shorthair was also predominant (*n* = 45), followed by Scottish Fold (*n* = 3), American shorthair (*n* = 2), domestic longhair (*n* = 1), Exotic Shorthair (*n* = 4), mixed-breed (*n* = 3), Persian (*n* = 2), Ragdoll (*n* = 4), and breed not recorded (*n* = 3).

In order to assess the influence of the complications associated with CKD in this study, cats with CKD were identified as having proteinuria, hypertension, and anemia. Additionally, the cats taking or not taking iron supplements were also considered. There were 16 cats with proteinuria and 45 without proteinuria; 17 with hypertension and 40 without hypertension; 11 with anemia and 45 without anemia; and 12 cats receiving iron supplementation compared with 44 not receiving iron supplementation (Figure 1).

### 3.3 Urine GPX4 levels, UGCR and clinical parameters

**Figure 2:**
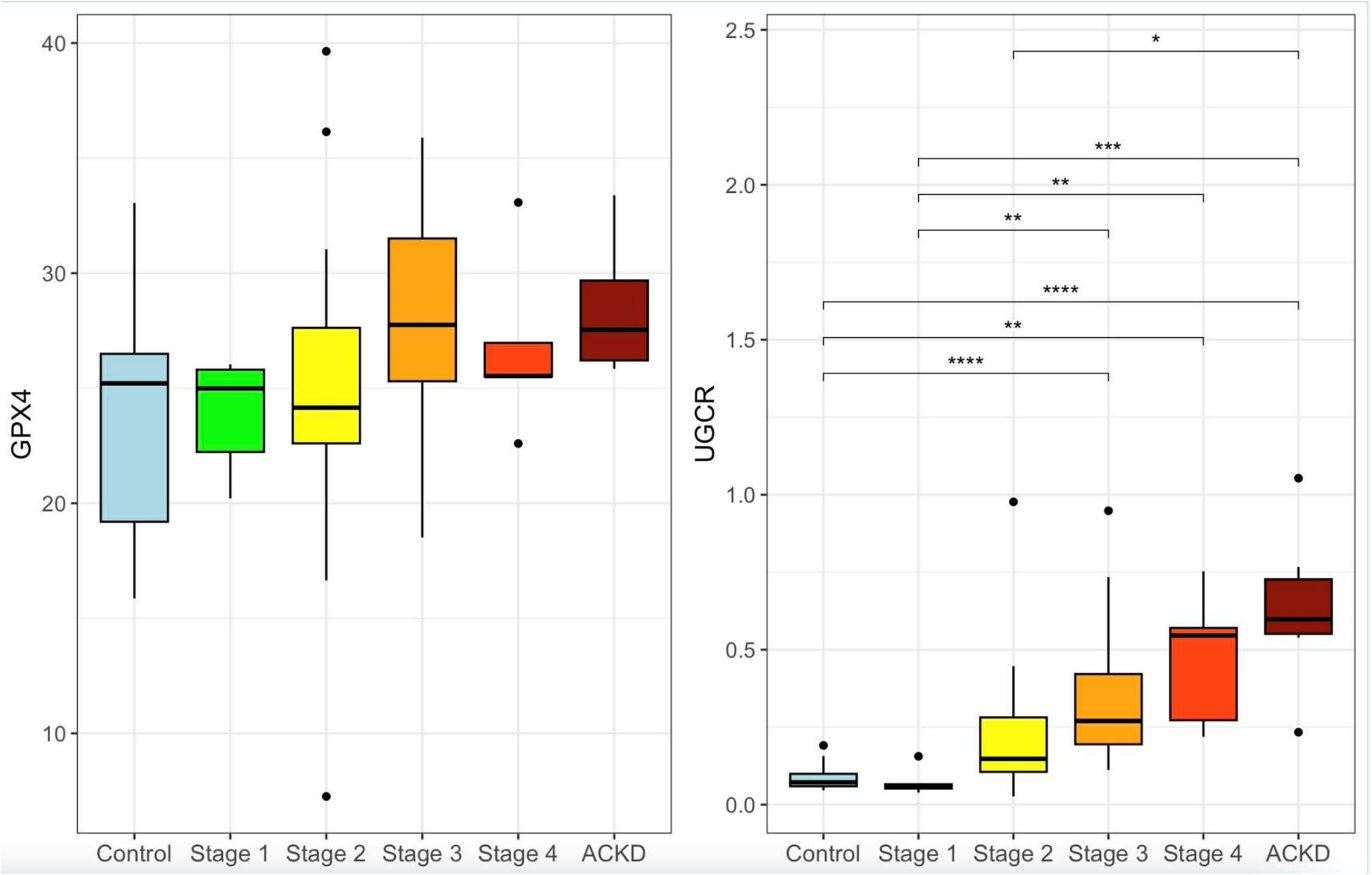
Urine *GPX4* levels (ng/mL) and *GPX4*-to-creatinine ratio (*UGCR*) (×10^−4^) in cats with different stages of chronic kidney disease (CKD) and acute-on-chronic kidney disease (ACKD). **(A)** Urine *GPX4* levels showed no significant differences among groups, although a mild decrease was observed in advanced CKD and ACKD. **(B)** *UGCR* increased progressively with CKD severity and was highest in ACKD (*p <* 0.05, *p <* 0.01, *p <* 0.001, *p <* 0.0001).

The urine GPX4 and UGCR measurements for the control group, the various IRIS CKD stages, and the ACKD group are shown in Figure 2. Urine GPX4 showed an increasing trend in the later stages of CKD, although the difference did not reach statistical significance. Of note, after adjustment for urine creatinine, UGCR rose significantly in parallel with a decline in renal function thus achieving a markedly elevated level during the stages 3 and 4 as well as when ACKD was present.

For further analysis, stages 1 and 2 of CKD were grouped together as early stage CKD, while stages 3 and 4 were grouped together as late stage CKD. The results for GPX4, UGCR, and various relevant clinical parameters across the control, early, and late CKD groups are summarized in Table 1. Compared with the control group (urine GPX4, median [IQR]: 25.21 [18.99–26.91]; UGCR: 0.072 [0.057–0.101] × 10^−4^) and the early-stage group (urine GPX4: 24.31 [22.00–24.07]; UGCR: 0.134 [0.070–0.260] × 10^−4^), both urine GPX4 (26.89 [25.11–31.66]) and UGCR (0.271 [0.197–0.457] × 10^−4^) were significantly increased in cats with late-stage CKD (*p* = 0.011) and ACKD group (*p* < 0.001), respectively.

**Table 1:** Parameters of interest and clinical characteristics of the control group, early CKD group, and late CKD group.

Parameters	Control (*N* = 15)	CKD groups (*N* = 61)				*p* value
		Early-stage CKD^a^ (IRIS stages 1–2; *N* = 33)		Late-stage CKD^b^ (IRIS stages 3–4; *N* = 28)		
GPX4 (ng/mL)	25.21 (18.99–26.91)	24.31 (22.00–24.07)	*N* = 33	26.89^c,d^ (25.11–31.66)	*N* = 28	0.011
UGCR (×10^−4^)	0.072 (0.057–0.101)	0.134 (0.070–0.260)	*N* = 33	0.271^c,d^ (0.197–0.457)	*N* = 28	<0.001
Age (years)	1.7 (1.0–6.0)	8.0 (4.0–12.0) c	*N* = 33	13.5^c^ (7.5–15.5)	*N* = 28	<0.001
Gender (% Female)	73.3	48.6	*N* = 33	42.9	*N* = 28	0.148
BUN (mg/dL)	21 (20–28)	35 (25–48)	*N* = 33	42^c,d^ (35.5–60.5)	*N* = 28	<0.001
Creatinine (mg/dL)	1.5 (1.3–1.6)	2.1 ^c^ (1.7–2.4)	*N* = 33	3.5^c,d^ (3.3–4.3)	*N* = 28	<0.001
Hematocrit (%)	37.2 (33.6–45.5)	39.3 (36.2–43.2)	*N* = 33	36.6 (28.6–42.0)	*N* = 28	0.092
Hemoglobin (g/L)	13.5 (11.5–15.7)	13.4 (12.5–14.5)	*N* = 33	12.5 (9.85–14.2)	*N* = 28	0.073
RBC (10^6^/μL)	8.59 (6.92–9.51)	8.32 (7.65–9.22)	*N* = 33	7.7 (6.27–8.81)	*N* = 28	0.036
MCV (fl)	46.7 (42.2–51.4)	34.2 (33.2–34.8)	*N* = 33	34.3 (32.8–35.2)	*N* = 28	0.987
MCHC (g/dL)	34.1 (33.7–35.7)	34.2 (33.2–34.8)	*N* = 33	34.3 (32.8–35.2)	*N* = 28	0.890
PLT (K/μL)	305 (235–377)	271 (220–356)	*N* = 33	273 (189–390)	*N* = 28	0.868
WBC (/μL)	7,500 (4,800–9,000)	7,000 (5,600–9,200)	*N* = 33	10,000^c,d^ (6,950–11,250)	*N* = 28	0.046
Albumin (g/dL)	3.6 (3.3–3.8)	3.2 (3.1–3.6)	*N* = 30	3.3 (2.9–3.6)	*N* = 23	0.058
Phosphate (mg/dL)	-	4.2 (3.6–4.6)	*N* = 24	4.5 (4.0–7.1)	*N* = 28	0.034
Sodium (mEq/L)	155.7 (154.7–157.6)	154.4 (153.4–156.3)	*N* = 33	155.5 (153.6–156.9)	*N* = 28	0.101
Potassium (mEq/L)	3.80 (3.63–3.87)	3.75 (3.51–3.90)	*N* = 33	3.90 (3.60–4.28)	*N* = 28	0.306
Chloride (mEq/L)	115.7 (114.4–117.7)	116.9 (114.9–118.6)	*N* = 33	116.8 (115.7–119.1)	*N* = 28	0.607
ALT (U/L)	43.0 (34.0–55.0)	45.0 (34.0–64.3)	*N* = 20	42.5 (30.3–150.3)	*N* = 10	0.912
USG	1.046 (1.041–1.053)	1.021 ^c^ (1.013–1.035)	*N* = 33	1.012^c,d^ (1.009–1.017)	*N* = 28	<0.001
UPC	0.05 (0.02–0.06)	0.07 (0.04–0.23)	*N* = 33	0.30^c^ (0.08–0.63)	*N* = 26	0.001
Urine pH	6.22 (5.93–6.91)	6.03 (5.86–6.36)	*N* = 33	5.93 (5.51–6.18)	*N* = 28	0.103

Numerous variables were tested with Kruskal Wallis Test and represented as the median (IQR). The post hoc analysis was Dunn’s test. Category variables were tested with *X*^2^ test.

^a^IRIS stage 1 and stage 2.

^b^IRIS stage 3 and stage 4.

^c^Achieved significance with the control group.

^d^Achieved significance with the early stage group.

GPX4, Glutathione peroxidase 4; UGCR, urine-GPX4-to-creatinine ratio; BUN, blood urea nitrogen; RBC, red blood cell; MCV, mean corpuscular volume; MCHC, mean corpuscular hemoglobin concentration; PLT, platelet count; WBC, white blood cell; ALT, alanine aminotransferase; USG, urinary specific gravity; UPC, urine protein-to-creatinine ratio.

Phosphate concentrations were not available for the control group because phosphate testing was not routinely performed in clinically healthy cats with normal renal indices and SDMA values.

Compared with the controls, cats in both early and late CKD stages were older (*p* < 0.001) and had higher BUN (*p* < 0.001) and plasma creatinine (*p* < 0.001) concentrations, along with a lower urine specific gravity (USG; *p* < 0.001) than the control group. Additionally, BUN (*p* < 0.001), plasma creatinine (*p* < 0.001), and phosphate (*p* = 0.034) and WBC (*p* = 0.033) were significantly higher in the late-stage group compared with the early-stage group.

### 3.4 Linear regression of UGCR and other clinical parameters

Simple regression analysis showed that UGCR was positively correlated with age (weak-to-moderate association), serum creatinine (strong association), WBC (weak association), phosphate (moderate association), and potassium (weak association), and was negatively correlated with hematocrit (moderate association). Among these, serum creatinine exhibited the strongest association (*R*^2^ = 0.46). A stepwise multiple regression analysis identified serum creatinine and WBC as independent variables related to UGCR, while the effects of age and other parameters were not significant (Table 2).

**Table 2:** Simple and multivariable linear regression analysis of clinical parameters against UGCR.

Parameters	Simple regression analysis			Stepwise multiple regression analysis	
	Adjusted *β*	*p* value	*R* ^2^	Adjusted *β*	*p* value
Age (years)	0.432	<0.001	0.19	0.199	0.065
Creatinine (mg/dL)	0.675	<0.001	0.46	0.557	<0.001
Hematocrit (%)	−0.505	<0.001	0.26		
WBC (/μL)	0.416	<0.001	0.17	0.259	0.019
Phosphate (mg/dL)	0.472	<0.001	0.22		
Potassium (mEq/L)	0.312	0.006	0.097		

GPX4, Glutathione peroxidase 4; UGCR, urine-GPX4-to-creatinine ratio; WBC, white blood cell.

### 3.5 Subgroup analysis

**Figure 3:**
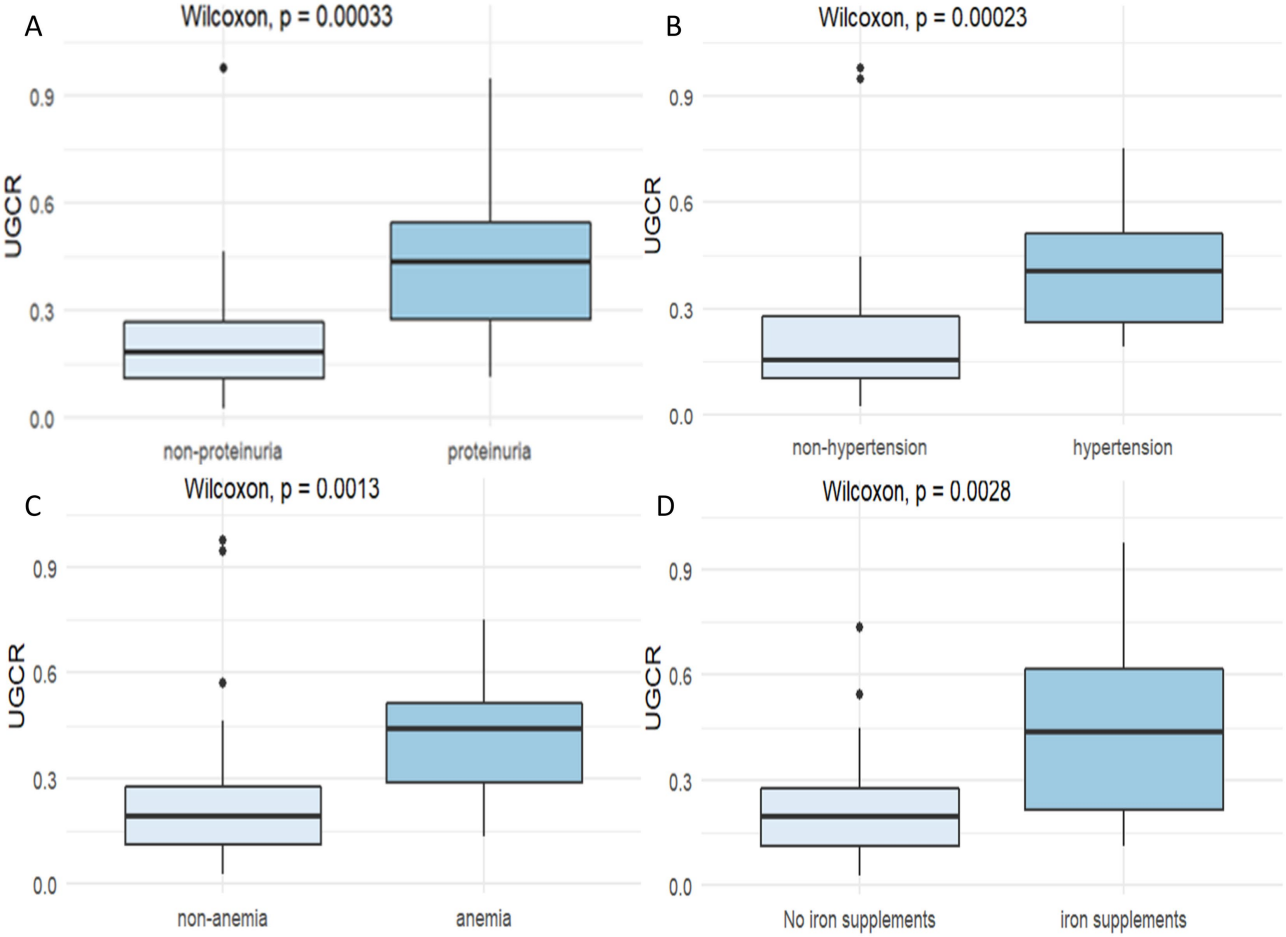
Box-and-whisker plots of the urine GPX4-to-creatinine ratio (UGCR) (×10^−4^) in the subgroups of cats with CKD. **(A)** Proteinuric and non-proteinuric groups. **(B)** Hypertensive and nonhypertensive. **(C)** Anemic and non-anemic groups. **(D)** Cats receiving and not receiving iron supplementation. Boxes represent the interquartile range (IQR), the horizontal line within each box indicates the median, and whiskers denote the range. Statistical comparisons between two groups were performed using the Mann–Whitney U test. GPX4, glutathione peroxidase 4; UGCR, urine GPX4-to-creatinine ratio.

UGCR was significantly higher in the proteinuric group compared to the non-proteinuric group (*p* < 0.001). Similarly, cats with hypertension exhibited markedly elevated UGCR compared with the non-hypertension group (*p* < 0.001). UGCR was also higher in anemic cats than in non-anemic cats (*p* = 0.001). Additionally, UGCR was significantly increased in cats with CKD who had been receiving iron supplementation for at least 1 month (*p* = 0.003) (Figure 3). To evaluate whether CKD stage influenced these findings, cats in both the anemia and non-anemia groups were further classified into the early and late stages of CKD. In the late-stage group, UGCR remained significantly higher in cats with anemia (*p* = 0.038), while in the early-stage group, UGCR showed a non-significant upward trend (*p* = 0.067) (Supplementary Figure S2).

### 3.6 Assessment of progression

In the cats with CKD that progressed within a 180-day follow-up period, UGCR was significantly higher in the progression group (*p* = 0.023) compared to the non-progression group. The clinical parameters of the progression and non-progression groups are summarized in Table 3. BUN (*p* = 0.001), plasma creatinine (*p* = 0.001), phosphate (*p* = 0.004), potassium (*p* = 0.012), and UPC (*p* = *0*.004) were significantly higher in the progression group. Additionally, in the progression group, albumin (*p* = 0.02), hematocrit (*p* = 0.007), hemoglobin (*p* = 0.01), and RBC count (*p* = 0.009) were significantly lower than in the non-progression group. However, in most cases, the hematocrit, hemoglobin, and RBC count were within the reference ranges for both groups.

**Table 3:** Parameters of interest and clinical characteristics of progression and non-progression groups.

Parameters	Non-progression (*N* = 33)		Progression (*N* = 15)		*p* value
GPX4 (ng/mL)	25.71 [22.85–29.9]	*N* = 33	26.82 [22.6–30.7]	*N* = 15	0.764
UGCR (×10^−4^)	0.176 [0.105–0.363]	*N* = 33	0.27 [0.219–0.546]	*N* = 15	0.023
Age (years)	9 [5–14]	*N* = 33	13 [8–15]	*N* = 15	0.378
BUN (mg/dL)	22.5 [17.4–30]	*N* = 33	60 [41–103]	*N* = 15	0.001
Creatinine (mg/dL)	2.4 [1.8–3.3]	*N* = 33	3.7 [2.9–7.6]	*N* = 15	0.001
Hematocrit (%)	40.2 [36.7–43.9]	*N* = 31	30.1 [24–39.1]	*N* = 14	0.005
Hemoglobin (g/L)	13.7[12.2–14.9]	*N* = 31	10.3[8.3–14]	*N* = 14	0.008
RBC (10^6^/μL)	8.39 [7.45–9.4]	*N* = 31	6.64 [5.57–8.36]	*N* = 14	0.006
WBC (/μL)	8,100 [5,850–10,425]	*N* = 31	8,600 [7,100–11,000]	*N* = 14	0.608
Albumin (g/dL)	3.2 [3.1–3.7]	*N* = 22	3.1 [2.8–3.5]	*N* = 12	0.026
Phosphate (mg/dL)	4.25 [3.7–4.63]	*N* = 22	6.7 [5.1–9.3]	*N* = 12	0.005
Sodium (mEq/L)	154.7 (153.4–156.4)	*N* = 22	155.3 (153.2–156.6)	*N* = 12	0.818
Potassium (mEq/L)	3.71 [3.47–3.9]	*N* = 22	4.39 [3.88–4.7]	*N* = 12	0.009
Chloride (mEq/L)	117.1 [114.9–118.6]	*N* = 22	117.05 [113.5–118.5]	*N* = 12	0.0.765
UPC	0.13 (0.05–0.35)	*N* = 31	0.51 (0.2–1.96)	*N* = 14	0.005

GPX4, Glutathione peroxidase 4; UGCR, urine-GPX4-to-creatinine ratio; BUN, blood urea nitrogen; RBC, red blood cell; MCV, mean corpuscular volume; MCHC, mean corpuscular hemoglobin concentration; PLT, platelet count; WBC, white blood cell; ALT, alanine aminotransferase; USG, urinary specific gravity; UPC, urine protein-to-creatinine ratio.

**Figure 4:**
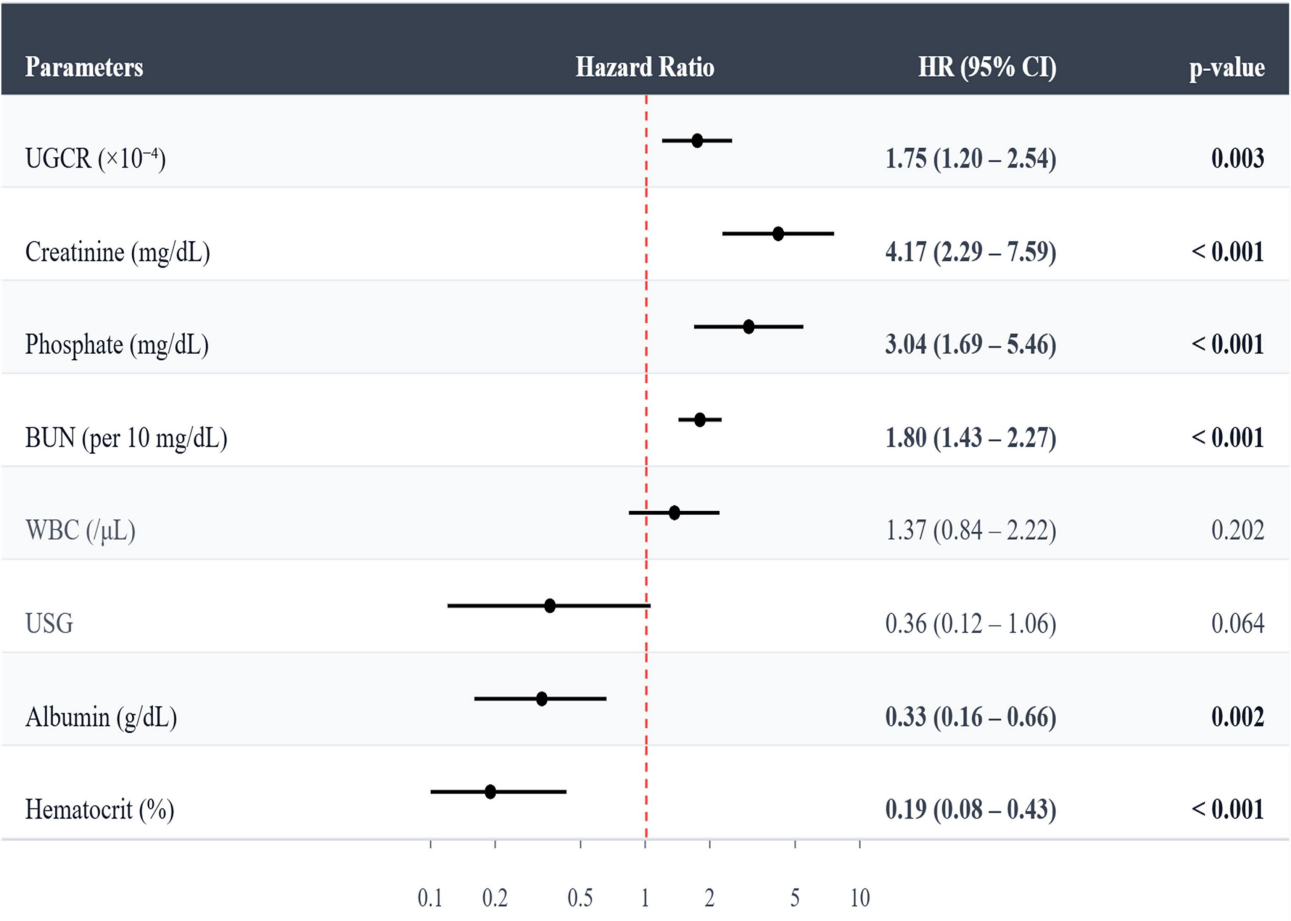
Univariable Cox proportional hazards models for predictors of CKD progression. 95% CI, 95% confidence interval; CKD, chronic kidney disease; UGCR, urine-GPX4-to-creatinine ratio; USG, urinary specific gravity; BUN, blood urea nitrogen; WBC, white blood cell count.

Elevated UGCR was significantly associated with an increased risk of CKD progression (Hazard ratio [HR], 1.75; 95% CI, 1.20–2.54; *p* = 0.003). Higher BUN (HR, 1.80; 95% CI, 1.43–2.27; *p* < 0.001), serum creatinine (HR, 4.17; 95% CI, 2.29–7.59; *p* < 0.001), and phosphate (HR, 3.04; 95% CI, 1.69–5.46; *p* < 0.001) were also positively associated with CKD progression. In contrast, lower albumin (HR, 0.33; 95% CI, 0.16–0.66; *p* = 0.002) and hematocrit (HR, 0.19; 95% CI, 0.08–0.43; *p* < 0.001) were negatively associated with progression risk (Figure 4).

ROC analysis demonstrated a modest but statistically significant discriminative performance of UGCR for CKD progression, with an AUROC of 0.707 (standard error 0.075, 95% CI 0.560–0.854, *p* = 0.023) (Supplementary Figure S3). Given the time-dependent nature of CKD progression, further prognostic evaluation focused on survival-based analyses.

**Figure 5:**
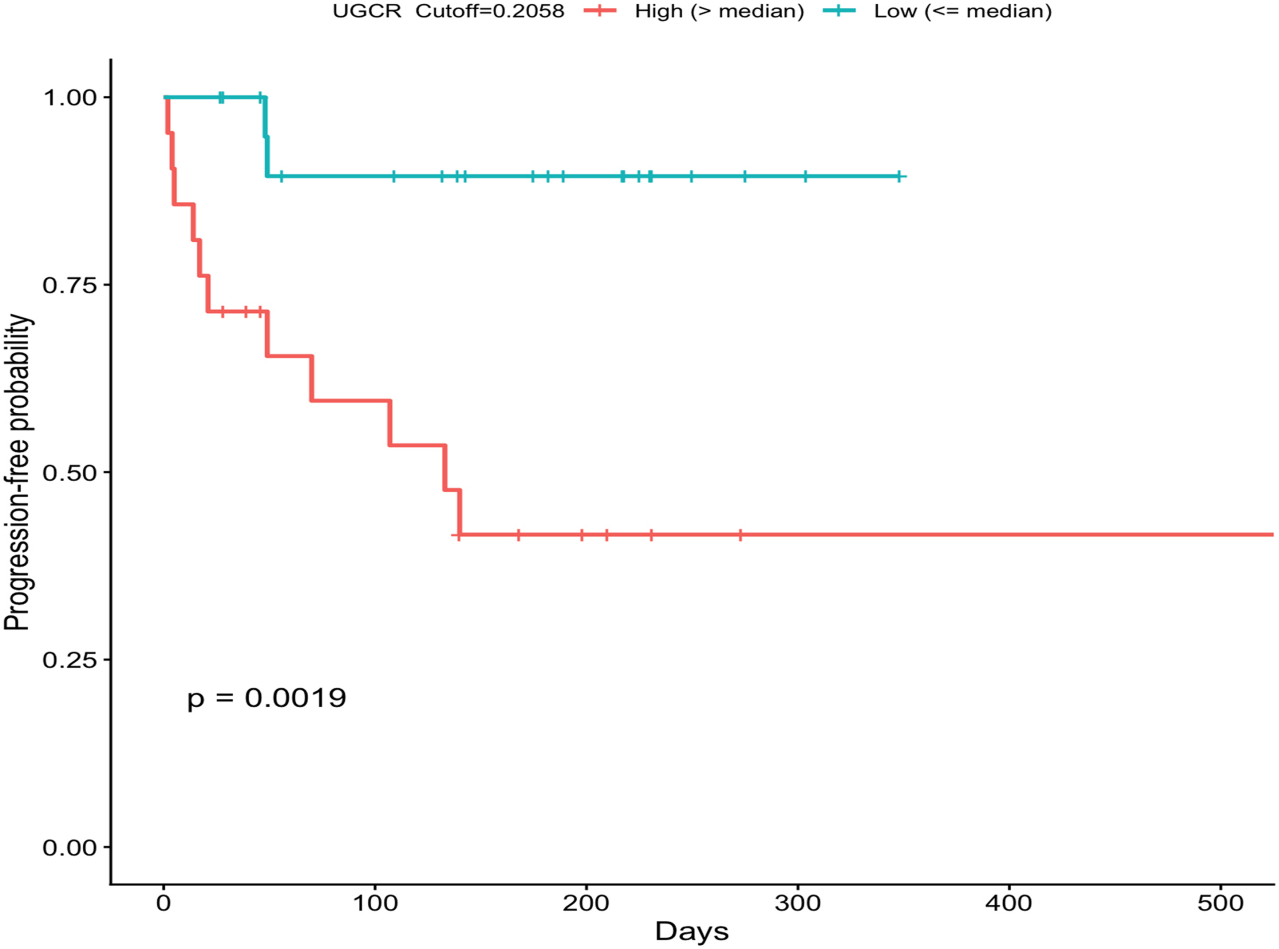
Kaplan–Meier curves for the time period to the progression of CKD in cats with UGCR ≤ 0.2058 × 10^−4^ (green line) and >0.2058 × 10^−4^ (red line); UGCR, urine-GPX4-to-creatinine ratio.

A UGCR threshold of 0.2058 was applied to stratify cats into low and high UGCR groups. Kaplan–Meier survival analysis revealed a significant difference in the progression-free interval between the two groups (log-rank *p* = 0.0019), with cats in the high UGCR group showing a shorter time to progression (Figure 5).

## 4 Discussion

To the best of our knowledge, this is the first study to investigate the relationship between urine GPX4 and the clinical parameters of CKD in cats. We observed that there was a significant increase in UGCR as renal function deteriorated at the late stages of CKD in cats, and that this was correlated with serum creatinine concentration. It is important to note that—this trend differs from human studies, which have reported reduced GPX4 expression in renal tissue biopsies of individuals with CKD (16). A similar reduction in renal GPX4 has also been documented in mouse models of fibrotic kidney disease (16–18). These studies suggest that ferroptosis occurs in CKD, as GPX4 is a primary regulator of ferroptosis, and this can lead to the release of large amounts of lipid peroxides, potentially causing changes in the phenotype of tubular epithelial cells (16, 23, 24). Importantly, prior studies have assessed GPX4 primarily in kidney tissue, whereas neither urine GPX4 detection nor GPX4’s relationship to tubular GPX4 has been previously investigated. Two hypotheses may be applied to explain the apparent discrepancy observed in relation to our study. First, as renal function declined, tubular epithelial injury and/or ferroptotic stress may lead to the leakage or shedding of intracellular GPX4 into urine, thus resulting in lower tissue expression but higher urinary levels. Therefore, elevated UGCR may reflect the combined effects of renal tubular injury and oxidative stress, and thus cannot be considered a specific indicator of ferroptosis in isolation. Second, species-specific differences in renal GPX4 biology cannot be excluded, as GPX4 expression has not been characterized in feline kidneys. Further studies are needed to elucidate the mechanisms underlying these important potential differences.

In the present study, UGCR was significantly higher in the proteinuric group compared to the non-proteinuric group. Previous research has shown that GPX4 plays a reno-protective role in tubular cells, with GPX4-null mice exhibiting marked increases in tubular cell death and proteinuria (12). Mice with neutrophil-specific GPX4 haploinsufficiency also developed a range of clinical features such as autoantibodies, neutropenia, and proteinuria, and treatment with a specific ferroptosis inhibitor significantly ameliorates disease severity (25). In humans, the gene expression pattern of GPX4 was also found to be significantly correlated with proteinuria in human focal segmental glomerulosclerosis samples (26). Assuming that urine GPX4 level rises as renal function declines, our findings are consistent with these previous studies.

Growing evidence suggests that an increase in reactive oxygen species (ROS) production and oxidative stress may be involved in the pathogenesis of hypertension (27, 28). Oxidative stress has been demonstrated to induce hypertension by activating the renin-angiotensin system (RAAS), promoting renal vasoconstriction, and impairing endothelial function (29). A genome-wide association study in a Spanish population identified GPX4 as a gene linked to CKD-related hypertension. GPX4 has been suggested to be involved in protecting cells against oxidative damage (30), which is consistent with its established protective role. However, this also needs further detailed investigation.

In the current study, UGCR was higher in CKD cats with hypertension compared to their normotensive counterparts. This finding again contradicts previous studies (16–18). However, these earlier studies measured GPX4 directly in kidney tissue, whereas our findings reflect GPX4 excreted into urine. This distinction raises the possibility that declining renal GPX4 content and increasing urine GPX4 levels may represent different facets of the same pathological process, namely that GPX4 depletion from tubular cells is accompanied by its leakage or shedding into urine during oxidative or ferroptotic injury. Nonetheless, species-specific differences between humans, rodents, and cats may also contribute to these divergent patterns, underscoring the need for further comparative studies.

In the present study, UGCR was significantly higher in CKD cats with anemia compared to those without anemia. Experimental evidence supports a link between GPX4 and erythropoiesis. This is because mice with hematopoietic cell–specific GPX4 deletion develop anemia that is characterized by an increased proportion of erythroid precursor cells and reticulocytes (31). Subsequent studies have demonstrated that GPX4 is essential for preventing anemia by inhibiting receptor-interacting protein kinase-3 (RIP3)–dependent necroptosis in erythroid precursor cells (32). These findings highlight the essential role of GPX4 in regulating the survival and maturation of erythroid cells. To date, however, direct evidence relating GPX4 to CKD-related anemia remains limited. Our results are the first to demonstrate an association between urine GPX4 levels and anemia in cats with CKD, although the underlying mechanism remains unclear. Furthermore, UGCR was significantly higher in cats receiving iron supplementation than in those without supplementation. When we separated the cohort into cats receiving iron supplementation and those not, the iron-supplemented group showed a less pronounced increase in UGCR across CKD stages, whereas the group without iron supplementation retained a clearer stage-dependent trend (Supplementary Figure S4). These findings suggest that iron supplementation may act as a potential confounder; however, the observed association between increasing UGCR and CKD severity is not solely attributable to iron supplementation. In a CKD mouse model, iron dextran treatment increased GPX4 transcription in kidney tissue (33), which suggests that iron therapy may influence GPX4 expression. Because anemic cats are more likely to receive iron supplements, both the underlying anemia and the effects of iron supplementation may contribute to the observed increase in urine GPX4 levels.

WBC count was identified as an independent variable associated with UGCR in our analysis. Previous studies have reported that GPX4 regulates neutrophil survival and inflammation by limiting ferroptosis (25) In addition, GPX4, together with vitamin E, protects hematopoietic stem and progenitor cells from lipid peroxidation, thereby maintaining white blood cell homeostasis (34). The positive association between WBC count and UGCR observed in this study suggests that leukocyte-related processes may influence urine GPX4 levels. However, the underlying mechanism remains unclear and warrants further investigation.

UGCR was highest in cats with ACKD, exceeding the levels observed in both the control group and all CKD subgroups. Previous studies have implicated ferroptosis in the pathogenesis of AKI (35, 36). Experimental studies have shown that mice with GPX4 deletion spontaneously develop AKI (12), whereas GPX4 upregulation prevents renal injury (37). Similarly, overexpression of GPX4 in diabetic rat kidneys was shown to alleviate ferroptosis-induced renal damage (38). These findings indicate that GPX4 plays a protective role in AKI. The markedly increased urine GPX4 levels observed in cats with ACKD may therefore reflect an active renal response involving GPX4 regulation. Nevertheless, the specific relationship between GPX4 in feline urine and AKI in feline CKD remains to be elucidated.

In cats with CKD that have progressed over a 180-day follow-up period, UGCR was significantly higher than in the non-progression group. ROC analysis of UGCR showed statistically significant results in relation to progression, even though the overall discriminative performance was modest. However, the univariate Cox proportional hazard model also showed that high UGCR was linked to a higher hazard ratio of progression; additionally, the survival curve also showed that the cats with high UGCR had a shorter progression time. Increased oxidative stress has been demonstrated to drive the progression of CKD (39). In human studies, GPX4, which is well known as a key antioxidant gene, has also been linked to CKD progression (30, 40). In an animal model of diabetic kidney disease, transcriptional profiling using a PCR array revealed that antioxidant therapy upregulated GPX4 expression, which in turn attenuated disease progression in diabetic rats (41).

Our study has several limitations. First, it was a single-center, retrospective analysis, which may limit the generalizability of the findings to other clinical settings. Misclassification bias and missing data are also possible. Second, the number of cases, especially in the ACKD and advanced CKD groups, was relatively small, which may have reduced the statistical power and increased the possibility of a type II error. Third, due to the retrospective study design, breed information was missing for a subset of cases. While this precludes the evaluation of breed-specific effects, all included cats met rigorous clinical and diagnostic criteria, ensuring the reliability of the renal data. Nonetheless, the influence of breed as a potential confounding factor cannot be entirely ruled out. Additionally, a more detailed analysis of iron supplementation, including the route, dose, and duration of administration, may also be informative but was not feasible with the available sample size. Furthermore, the observational nature of the study precludes conclusions regarding causality between UGCR and disease progression. Additionally, the possibility of an age-related effect cannot be ruled out without age-matched controls. Lastly, the lack of renal biopsy prevented the pathological confirmation of kidney disease and of an assessment of GPX4 expression in kidney tissue.

In conclusion, UGCR was found to be closely linked to renal function and inflammation status in cats, and showed significant increases in late-stage CKD and even higher levels in those with acute-on-CKD. UGCR was also related to important clinical issues like anemia, proteinuria, and hypertension. These findings suggest that UGCR may serve as a valuable biomarker for assessing CKD severity and may have potential utility for the monitoring disease progression, thus helping clinical risk stratification and ongoing management of feline patients.

## Data Availability

The original contributions presented in the study are included in the article/Supplementary material, further inquiries can be directed to the corresponding author.

## Supplementary material

SUPPLEMENTARY FIGURE S1 Standard calibration curve for measuring feline urinary GPX4 concentrations by ELISA.

SUPPLEMENTARY FIGURE S2 Box-and-whisker plot of the urine GPX4-to-creatinine ratio (UGCR) in anemia and non-anemia groups, further stratified into early- and late-stage CKD groups.

SUPPLEMENTARY FIGURE S3 ROC curve of UGCR for predicting progression within the 180-day follow-up period. Sen: Sensitivity; Spe: specificity.

SUPPLEMENTARY FIGURE S4 Distribution of cats by CKD stage and iron supplementation status. Cats were categorized according to IRIS CKD stages 1–4 and further stratified by iron supplementation. The number of cats without versus with iron supplementation was as follows: stage 1 (5 vs 0), stage 2 (22 vs 3), stage 3 (15 vs 6), and stage 4 (2 vs 3).
