# Supplementary material for: Evaluation of urine glutathione peroxidase 4 in cats with chronic kidney disease
Source: Front Vet Sci. 2026 Jan 14;12:1756038. doi: 10.3389/fvets.2025.1756038 (PMC12846965; doi:10.3389/fvets.2025.1756038)
Supplement: Supplementary file 1 [file Data_Sheet_1.pdf]

Cat Glutathione Peroxidase 4 (GPX4) ELISA Kit

Catalog #: MBS093863

Lot: 05/2023

Intra-assay Precision (Precision within an assay): Three samples of known concentration were tested twenty times on one plate to assess intra-assay precision.

Inter-assay Precision (Precision between assays): Three samples of known concentration were tested in six separate assays to assess inter-assay precision.

	Intra-Assay Precision			Inter-Assay Precision		
Sample	1	2	3	1	2	3
n	20	20	20	6	6	6
Mean (ng/ml)	4.99	18.93	73.29	5.39	21.15	82.65
Standard deviation	0.23	0.88	3.44	0.32	0.93	3.50
CV (%)	4.6	4.7	4.7	6.0	4.4	4.2

MyBioSource

FOR RESEARCH USE ONLY. NOT FOR USE IN DIAGNOSTIC PROCEDURES.
